# Business Meetings

**Published:** 1888-12

**Authors:** 


### BUSINESS MEETINGS.

The two Associations held separate meetings for the transaction of the usual business, payment of dues, election of new members, necrological resolutions, elections, etc. But little special business was transacted by either Association. In both Associations resolutions were adopted and committees appointed to memorialize Congress with reference to the removal of the tax or duty on dental goods of all kinds.

A resolution was adopted in the American Association declaring it non-professional for a dentist to place on his card anything more than name, title and address.

In the Southern Association, the *status* of the International Tooth-Crown Co.'s suits for infringements, licenses, royalties, etc., was discussed at length, and arrangements made for securing a fund for defence in test cases.

